# Impact of pre-OP independence in patients with limited brain metastases on long-term survival

**DOI:** 10.1186/s12885-020-07459-z

**Published:** 2020-10-08

**Authors:** Annalen Bleckmann, Benjamin Kirchner, Manuel Nietert, Micha Peeck, Marko Balkenhol, Daniela Egert, T. Veit Rohde, Tim Beißbarth, Tobias Pukrop

**Affiliations:** 1 Clinic for Hematology/Medical Oncology, University Medical Center Göttingen, 37099 Göttingen, Germany; 2 Dept. of Medical Bioinformatics, University Medical Center Göttingen, 37099 Göttingen, Germany; 3 Medical Clinic A, Haematology, Haemostasiology, Oncology and Pulmonology, University Hospital Münster, 48149 Münster, Germany; 4 G-CCC, University Medical Center Göttingen, 37099 Göttingen, Germany; 5 Department of Neurosurgery, University Medical Center Göttingen, 37099 Göttingen, Germany; 6 Clinic for Internal Medicine III, Hematology and Medical Oncology, University Regensburg, 93053 Regensburg, Germany

**Keywords:** Brain metastases, Activities of daily living, Independence, Surgery, Katz-index, GPA

## Abstract

**Background:**

Brain metastasis represents a major complication with a significantly shorter overall survival of many oncological diseases, in particular of lung cancer, breast cancer and malignant melanoma patients. However, despite the poor prognosis, sometimes clinical decision-making, between on the one hand not to harm the patient and on the other hand not withholding a potential therapeutic option, is very challenging. Thus the aim of this retrospective study was to compare various scores, including scores for activities of daily living (ADL) before resection of brain metastases and to analyse their impact on survival.

**Methods:**

Our single institution retrospective patient cohort (*N* = 100) with a median age of 63.6 years, which had all undergone resection of one or more brain metastases, was categorized using the original patient files. The cohort includes 52 patients with lung cancer, 27 patients with breast cancer, 8 patients with colorectal carcinoma and 13 patients with kidney cancer. To categorize, we used different score systems which were capable to evaluate the patient in relation to self-sufficiency, activity and self-determination as part of ADL. The retrospective analysis includes the ECOG-Status, Karnofsky-Index, Barthel-Index, ASA-Classification and Katz-Index. Pre-processing and the analysis of the data was implemented using KNIME, where we used the R-plugin nodes to perform the final statistical tests with R.

**Results:**

Our analysis reveals that most of the ADL scores we tested are able to give a reliable prediction on overall survival after brain metastasis surgery. The survival rates decrease significantly with a lower score in all tested score systems, with the exception of the ASA-Risk score. In particular, the Katz Index < 6 was identified to have a significant correlation with a lower cancer specific survival (CSS) (HR 3.33, 95%-CI [2.17–5.00]; *p*-Value = 9.6*10^− 9^), which is easy to use and has reproducible measurements.

**Conclusions:**

Pre-operative independence assessment by indices of ADL represents a predictor for overall survival after resection of brain metastases. Especially the easily, objectively and rapidly applicable Katz-Score is a very helpful tool to assess the pre-operative status, which could be additionally included in clinical decision making in daily practice.

## Background

Brain metastases represent the most common intracranial tumor and are generally associated with a limited prognosis and a reduced median overall survival (OS). The OS of untreated patients is only a few weeks to months [9].

The various primary cancers differ significantly in their frequency developing brain metastases. Most frequently brain metastases are formed by non-small cell lung cancer (NSCLC) and breast cancer, which cause 50% and 15–20% of all brain metastases, followed by malignant melanoma and renal cell carcinoma. Gastrointestinal and genitourinary tumors are only responsible for 5–10% of all brain metastases [9].

Overall, it seems that the incidence of brain metastases is increasing. Some possible explanations have been proposed: First of all, considering the demographic shift to an older population, the overall burden of different primary malignancies and therefore brain metastases originating from these is increasing. Second, the improved diagnostic procedures are capable to detect cerebral metastases in very early stages [20]. Moreover, better treatment strategies of the primary tumor and extracranial metastases lead to an improved OS, which subsequently increases the risk of developing brain metastases over time. Altogether, these factors point towards a challenge to our health system with increasing numbers of comorbid patients with brain metastasis in the future where improved tools for clinical decision making are needed.

Since most of the patients suffering from brain metastases were excluded from clinical trials in the past, there are limited tools to determine the risk and life expectancy before neurosurgical treatment. The first prognostic tool was the recursive partition analysis (RPA) of the Radiation Therapy Oncology Group (RTOG), which provided three prognostic classes based on patients from different trials undergoing whole-brain irradiation [8]. Four factors were included in this score: the Karnofsky Performance Status (KPS), age, controlled extracranial disease and extracranial metastases. A subsequent trial revealed that WBRT (whole brain radiation therapy) and stereotactic boost treatment in patients with up to three brain metastases improved functional autonomy (KPS) in all patients and survival in patients with a single unresectable brain metastasis [2]. Therefore in 2008 a new score, the Graded Prognostic Assessment (GPA), has been suggested [26]. Considering the data Andrews et al. provided, this assessment includes the number of cerebral metastases instead of the condition of controlled extracranial disease. The GPA does appear to be as prognostic as the RPA and is less subjective, because the RPA requires assessment of whether the primary disease is controlled. It is also more quantitative, easier to use and remember [26]. Moreover, the GPA score was more refined and nowadays almost each tumor entity has its own GPA score. These scores are also constantly updated. The newest scores even included molecular markers in NSCLC, where e.g. EGFR- or ALK-mutations are incorporated in the new molecular lung GPA [28, 29] (mollungGPA) [29]. However, independent of the primary tumor, one decisive factor in all GPA scores still is the KPS, and in some tumors, e.g. in gastrointestinal tumors [25], it is even the most important factor. Interestingly, this is the only factor which still is a subjective estimate, while all others like age, number of metastases, molecular markers and extra cerebral tumor manifestations are objectively evaluable parameters. Thus, in this study, we aimed to search for an alternative criterion, which is more objectively assessable and evaluated several scores for activities of daily living (ADL), which are already routinely used in geriatric and other clinical assessments. To shed more light on this subject, we aimed to determine the impact of independence on OS in a single-centre cohort of patients undergoing resection of brain metastases. Therefore, we collected data concerning scores of ADL, a widely accepted measure of dependency in elderly individuals ([12, 16].

## Methods

### Study cohort

All patients except patients with malignant melanoma, which had undergone a resection of brain metastasis at the department of neurosurgery at the University Medical Center Göttingen (UMG) between 08/2003 and 11/2013, were included in the study. The expected heterogeneity of primary cancers ranged from NSCLC, breast cancer, colorectal cancer to renal cancer. Metastases from patients with malignant melanoma were not assessed because their pathway of infiltration is different to the other primary tumors [4]. All histological analyses were performed by the department of Neuropathology at the UMG.

The patient cohort was characterized in terms of demographics, clinical baseline data and treatment regimens. Follow-up examinations were performed according to individual physicians’ discretion and data were obtained either from the local clinical cancer registry or treating physicians. OS after primary neurosurgical treatment was defined as the interval between the surgical resection of the brain metastasis and death, which was cancer-related in all cases. Last follow-up was 02/2016.

The data on ADL-scores was assessed from different sources: i) the original patients file archived by the department of Neurosurgery, ii) several clinical documentation sources/programs, which consist of files from the pathology department of the UMG, the clinical information and the tumor documentation system from the UMG, data from the registry office and primary care physicians.

Patients with insufficient documentation of ADL at the timepoint of the surgical intervention were not included. All patients where the origin of brain metastasis could not be uniquely identified by pathological assessment were excluded from this study. Likewise, patients where the cancer’s primary site wasn’t clearly identified by pathological analysis were excluded.

The applied scores were the Eastern Cooperative Oncology Group (ECOG) performance status [18], the Karnofsky performance status [11], the ASA-Classification [7, 23] as well as the ADL scores Barthel Index [16] and Katz Index [12]. To categorize, we used these different score systems which are capable to evaluate the patient in relation to self-sufficiency, activity and self-determination as part of activities of daily living. We also compared the sub-items of the different Score-Systems (e.g. dressing, eating). Katz and Barthel Score-Systems are mostly used in nursing documentation to measure the patient’s independence and performance status. All of them were retrospectively applied by examination of the above-mentioned data sources. The Katz index covers independence in feeding, bathing, dressing, transferring, toilet use and urinary continence as well as impairment in ambulation (inability to walk independently, use of walking sticks permitted) or cognition (previous diagnosis of dementia or signs of temporal, topographical or personal disorientation on admission). The Barthel Index addresses the following items: Presence or absence of fecal and urinary incontinence, help needed with grooming, toilet use, feeding, transfers, walking, dressing, climbing stairs and bathing.

### Pre-processing and statistical analysis

Score parameter distribution was correlated with clinicopathological parameters (Hypertension, Count of metastases and so on). The workflow for the pre-processing and the analysis of the data was implemented using KNIME 3.2.1 [3]. KNIME nodes were mostly used for the pre-processing of the data, while we used the R-plugin nodes in KNIME to perform the final statistical tests with R version 3.0.2 [21]. The global significance level was set to α = 5%. For comparisons of continuous data we used the Pearson’s correlation coefficient (r). If the data was skewed we used the non-parametric, rank based correlation coefficient (tau) according to Kendall [13]. For comparisons of two continuous data distributions we used the Wilcoxon rank sum test; paired where applicable [14]. In case of three or more different distribution samples we used the Kruskal-Wallis rank sum test for the comparison [15]. In case of count data we used the Fishers Exact test or Chi-Square test depending on the available number of samples for the comparison.

The impact of the ‘Scores” and ‘clinical parameters’ on cancer specific survival (CSS) was determined using Kaplan-Meier analysis and assessed for statistical significance using the log rank test and where applicable for the continuous data values using a Cox proportional hazard model [6]. The survival analysis was performed using the R package survival and for feature selection the step function in backward-mode from the stats package. For Sammon mapping [24] we used the KNIME node ‘MDS (Distmatrix)’ to project the data in conjunction with R to create the plot.

## Results

### Characterization of patient cohort

The patient cohort was characterized in terms of demographics, clinical baseline data, and treatment concepts according to the parameters listed in Table 1 and Supplementary Table 1.

**Table 1:** Univariate analysis of clinicopathological baseline data affecting survival: Patient cohort was characterized according to listed parameters in the first column. Type of classification and distribution within the cohort as well as impact on survival including *P*-Value (logrank) is given for each parameter. Bold and underlined *P*-Values are meant to highlight those below 0.05. NA = cases where not for all patients baseline data was available

Parameter:	Classification	Distribution	Impact on cancer specific survival (CSS) Hazard ratio [95%-CI]	P-Value (logrank)
**Age**	< 63,6 > = 63.6 Min 34, Max 87 Mean 62	50% (50/100) 50% (50/100)	Age > 63.6: 2.48 95% CI [1.62–3.81]	**1.8 × 10**^**− 5**^
**Sex**	Male Female	43% (43/100) 57% (57/100)	Gender male: 1.62 95% CI [1.07–2.46]	**0.022**
**Number of cerebral metastases**	Solitary > 1	44% (44/100) 56% (56/100)	Multiple metastases: 1.53 95% CI [1–2.24]	**0.048**
**Extracranial metastases at diagnosis of brain metastasis** NA = 1	Yes No	33% (33/100) 66% (66/100)	Present at surgery: 1.34 95% CI [0.86–2.07]	0.19
**Chemotherapy (CT) after brain surgery** NA = 7	No Yes	50% (50/100) 43% (43/100)	No postoperative CT: 1.19 95% CI [0.77–1.83]	0.43
**Radiotherapy (RT) after brain surgery** NA = 1	No Yes	70% (70/100) 29% (29/100)	No postoperative RT: 1.75 95% CI [1.1–2.78]	**0.017**
**Scores**				
**Katz Index (median)**	< 6 =6	47% (47/100) 53% (53/100)	Impairment (< 6): 3.29 95% CI [2.13–5.07]	**9.6 × 10**^**−9**^
**Barthel Index (median)**	<=90 > 90	66% (66/100) 33% (34/100)	Impairment (<=90): 2.7 95% CI [1.69–4.33]	**1.5 × 10**^**− 5**^
**Karnofsky Performance Score (median)**	< 60 > = 60	33% (33/100) 67% (67/100)	Low Score (< 60): 3.55 95% CI [2.24–5.64]	**1.4 × 10**^**− 8**^
**ECOG Scale (median)**	< 2 > = 2	36% (36/100) 64% (64/100)	Low Score (< 2): 2.74 95% CI [1.73–4.34]	**9 × 10**^**− 6**^
**ASA Classification (median)**	<=2 > 2	61% (61/100) 39% (39/100)	Low Score (< 2): 1.28 95% CI [0.84–1.95]	0.26

In total, 100 patients with different solid malignancies were included in this study. Fourteen patients had previously been excluded, 7 of which had insufficient nursing documentation and 7 had a not clearly identifiable primary tumor. The cohort includes 52 patients with brain metastases of NSCLC, 27 patients with breast cancer, 8 patients with colorectal cancer and 13 patients with renal cell carcinoma. All patients were diagnosed with brain metastasis and underwent surgery at the median age of 63.6 years (ranging from 54.4 to 86.5 years). Age above the median age was shown to be significantly associated with decreased survival (*p* = 1.8 × 10^− 5^, HR = 2.48).

In total 57 female patients and 43 male patients were included, with a tendency to shorter survival in male patients (*p* = 0.022, HR = 1.62). Individual treatment strategies are summarized in Table 1. The OS was 6.41 months survival after resection of the brain metastasis. Survival was significantly shorter in the cohort with more than one metastasis in the brain (*p* = 0.048, HR = 1.53), but not significantly influenced by extracranial metastases. Furthermore the analysis of radiotherapy revealed a worse survival for patients, who did not receive radiotherapy (*p* = 0.017, HR = 1.75). On the other hand, omitting postoperative chemotherapy had no significant impact (*p* = 0.43; HR = 1.19). As shown in Supplementary Table 1 the patient cohort was also characterized in terms of comorbidities. Except for arterial hypertension (*p* = 0.003, HR = 1.91), there was no significant impact on survival on any of the listed parameters.

### Impact of scores on survival

**Fig. 1:**
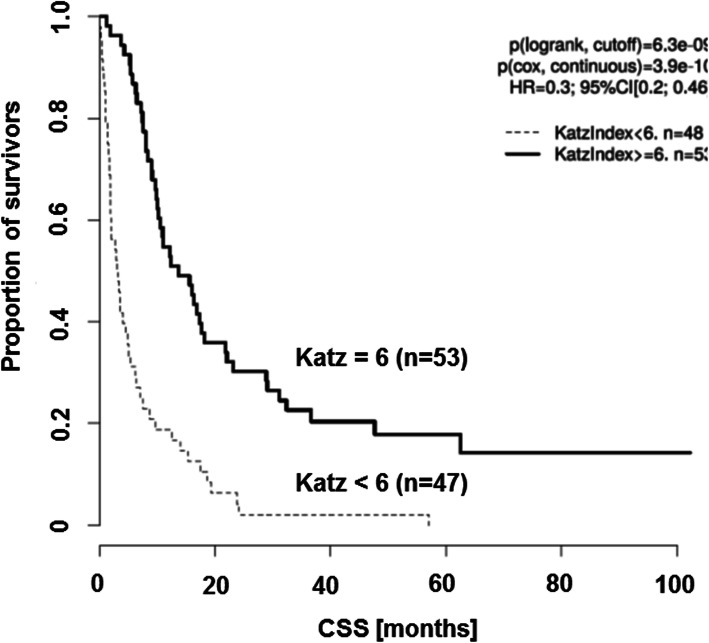
Kaplan-Meier curves illustrate that the Katz index has a significant impact (Cox proportional hazard ratio) on survival of brain metastasis patients. Cancer specific survival (CSS) is given in months

**Fig. 2:**
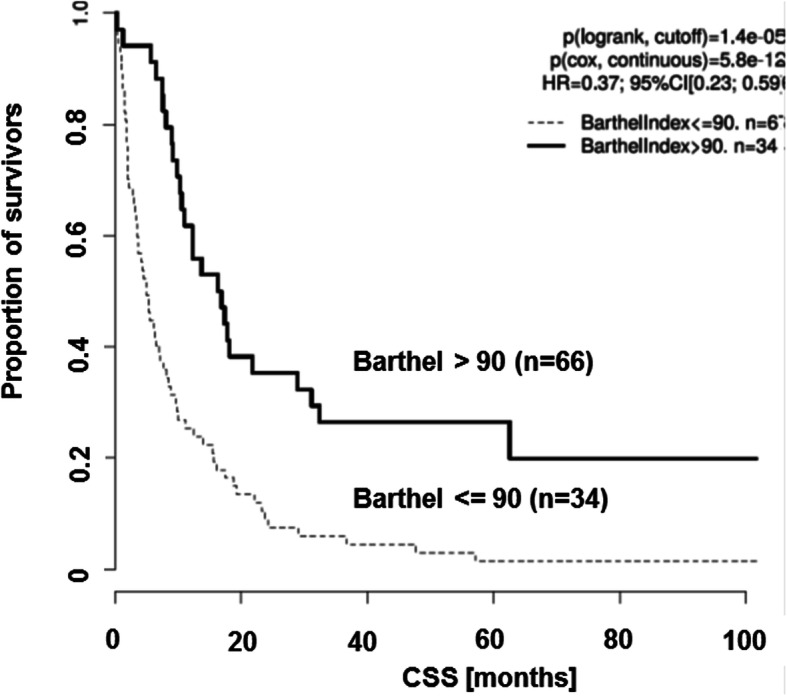
Kaplan-Meier curves illustrate that the Barthel index has a significant impact (Cox proportional hazard ratio) on survival of brain metastasis patients. Cancer specific survival (CSS) is given in months

The univariate analysis of the various scores and their impact on survival is displayed in the second half of Table 1. The ECOG Scale as well as the KPS revealed to be of relevance in CSS. Here, survival was significantly shorter in patients with low KPS (cutoff median 60; *p* = 1.4 × 10^− 4^; HR = 3.57) and high ECOG Scale (cutoff median 2; *p* = 9 × 10 ^− 6^; HR = 2.74). In contrast, the ASA Classification had no significant impact on survival (*p* = 0.26; HR = 1.28). Interestingly, the two addressed ADL indices exhibited a strong impact on survival for those patients, which did show a functional impairment before brain surgery (Figs. 1 and 2). Patients with any change in Katz index were assumed to be impaired, which led to significantly shorter survival (*p* = 9.6 × 10^− 9^; HR = 3.33; Fig. 1). This is in line with the results for the Barthel Index, where impairment was defined as a score < =90 (*p* = 1.5 × 10^− 5^; HR = 2.7; Fig. 2).

### Correlation of individual index parameters

**Fig. 3:**
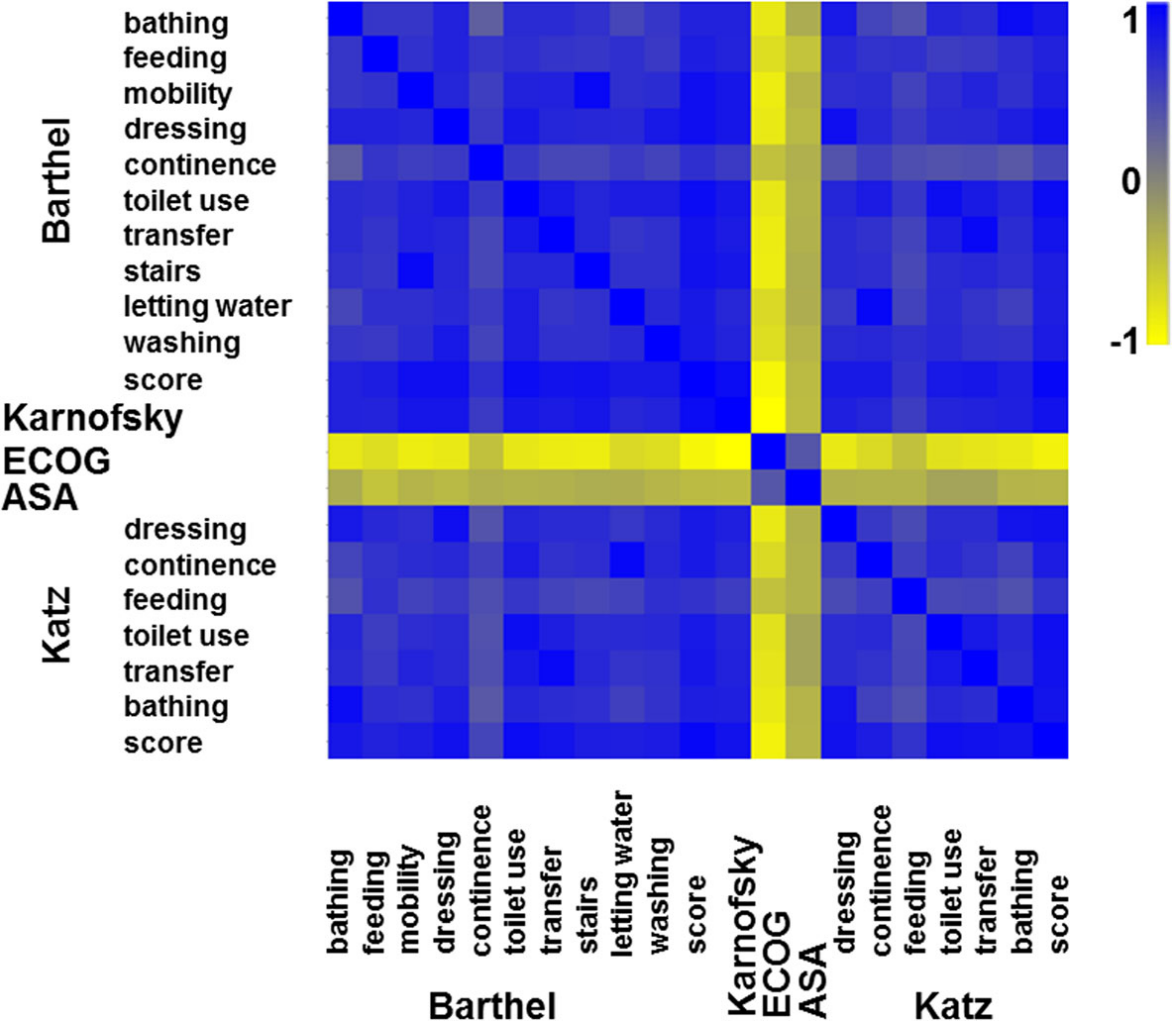
Heatmap based on correlations coefficients of the applied scores and index as well as their individual parameters. The coefficients from 1 to-1 are indicated in the scale bar. A high correlation between the two scores for ADL and their individual items as well as between ADL scores and the two performance scales could be seen

**Fig. 4:**
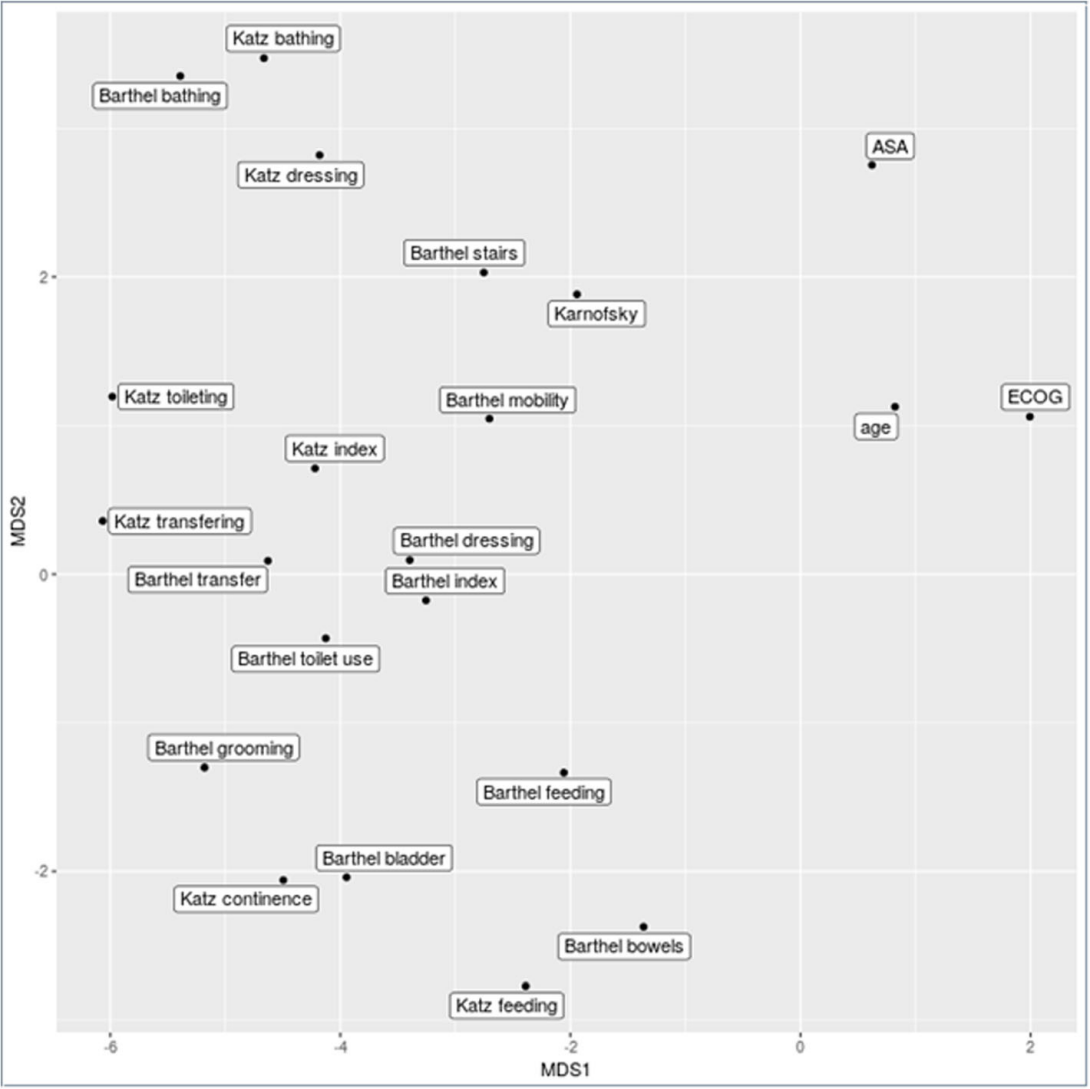
Multi Dimensional Scaling plot to preserve relative distances of data points relative to their respective distance from each other determined by their correlation distance matrix structure; Sammon mapping. The clustering of similar items from each score and the proximity of the two overall scores indicate a possible interchangeability of the two scores. The proximity of both scores to the KPS is also noteworthy as is the distinction of age as an independent parameter

In the next step, the different applied scores and indices as well as their individual parameters were correlated with each other (Fig. 3). As depicted, the different parameters are usually highly correlated. Both scores for ADL are therefore highly correlated with each other. The scores are also highly correlated to the individual performance scales (negatively for ECOG, where a low score, opposed to all other items, signifies a low performance). To address the question which of the parameters are most important, a feature selection of the items was performed. Here the consensus result was that the – from individual score parts – derived mixed model contained parts of Katz and Barthels alike. To investigate this further we performed a multi-dimensional scaling of the scores and indices (Fig. 4). Albeit the questionnaires differing slightly in the used language, and thus differences can be expected, the overall clustering of terms can be seen, e.g. see Fig. 4 for the Katz and Barthels positions of bathing, transfer and feeding. Katz and Barthels indices thus cover an overlapping feature space, which also leads to the relative close proximity of the summarized index. Due to the fact that the scores and indices show such a strong correlation, no further multivariate analysis was performed. But there is a distinction of age as an independent parameter. A median age of 63.6 years has been found to be significantly associated with a worse survival.

## Discussion

As previously mentioned, independent of the tumor entity the KPS estimation as performance status is still one feature included in all GPA scores. In this study we compared five performance scoring systems preceeding surgical removal of brain metastasis, including the KPS, to assess their impact as a single parameter on the individual prognosis of patients with brain metastases. Like Agarwal et al. and others we could underline that the KPS is a good predictor for OS among different entities (Low Score (< 60): HR 3.55 95% CI [2.24–5.64]). However, we also recognized that the KPS, when assessed independently of nursing documentation, retrospectively often doesn’t match the patients’ true independency shown in the nursing documentation. This result also fits our observations in clinical routine.

In this context it was highly interesting that the objectively evaluable Katz Index has the best prediction of the CSS in our univariate analysis, although just marginally (Low Score (< 6): HR 3.33 95% CI [2.17, 5.00]). This may be explained by the fact, that the six items of the Katz index, which are very easy to assess and objectively examinable parameters, might represent an at least as exact measure of performance in this patient population as does the KPS. The use of the Katz Index in this context is made even more appealing by the fact that the Katz index is often already well-established and routinely available in the nursing documentation. Thus, the Katz index could serve as a complimentary parameter besides the KPS to be used during the evaluation of the prognosis of brain metastasis patients. However, our study is retrospective and prospective data are still missing to test the predictive value of the Katz index. The Katz Index has already been prospectively shown to predict outcomes in other settings, e.g. in patients undergoing a transcatheter aortic valve replacement, where it was a powerful predictor of early and late outcome [19]. But the value of the Katz index, opposed to the KPS, is also doubtful in other settings, e.g. in patients undergoing radical cystectomy, where KPS was significantly correlated with complications at 30 and 90 days postoperatively while the Katz index was not [5]. Additionally, one might argue that the good correlation between the KPS and the Katz index here, as well as that of the Barthel with the ECOG [10] in palliative patients, might make it unnecessary to use more objective tools for the assessment of patients’ performance.

It is still remarkable that a loss of one of the fields of competence included in the Katz index is associated with a significantly worse survival. Even mild dependence therefore has a major adverse prognostic significance.

Whether a reversal of the functional deficit by corticosteroids, which are routinely used and have shown improvement of KPS [22] in the setting of brain metastasis, offsets the adverse prognostic effect cannot be answered by this study but would be an interesting area of further research.

Another issue is that deficits in basic ADL are common patients in cancer. A recent meta-analysis estimates these to be prevalent in about one-third of cancer patients [17]. Whether this instrument is therefore specific enough is debatable, but the patient population in this study with 47% exhibited an even higher fraction of deficits in ADL. An alternative to the assessment of ADL could be to include patient-reported functional outcome based on the EORTC QLQ C30 [1]. One study revealed that the EORTC QLQ C30 added prognostic value of a patient-reported functional outcome score in patients with NSCLC with brain metastases to patients just scored with KPS. This study included 140 patients with NSCLC. This study indicates, that the use of patient-reported performance status can provide the same prognostic information as KPS in patients of NSCLC with brain metastases.

The independent prognostic value of age is in accordance to age having been previously shown to be an independent prognostic parameter in the GPA of NSCLC and breast cancer [27], which constitute the majority of our samples. Taken together with the significant results in number of brain metastases and radiotherapy’s impact of survival, which are also incorporated into most GPAs [27], this underlines the soundness of this study’s data.

## Conclusion

In this study we demonstrate that the more objective Katz index could be used complimentary or maybe even as an alternative to KPS as part of the GPA scores. However, a combination of these scores has not yet been tested in a prospective cohort, but since the Katz index is often part of the nursing documentation, the use as an additional criterion in critical situations to the GPA result is uncomplicated and should lead to an even more complete picture of the individual patient.

## Supplementary information

**Additional file 1.**

## Data Availability

Data sharing not applicable to this article as no datasets were generated or analysed during the current study.

## Abbreviations

ADL: activities of daily living
CSS: cancer specific survival
ECOG: Eastern Cooperative Oncology Group
GPA: Graded Prognostic Assessment
KPS: Karnofsky performance scale
NSCLC: non-small cell lung cancer
OS: overall survival
RPA: recursive partition analysis
RTOG: Radiation Therapy Oncology Group
UMG: University Medical Center Göttingen
WBRT: Whole brain radiotherapy
