# Supplementary material for: Impact of pre-OP independence in patients with limited brain metastases on long-term survival
Source: BMC Cancer. 2020 Oct 8;20:973. doi: 10.1186/s12885-020-07459-z (PMC7545555; doi:10.1186/s12885-020-07459-z)
Supplement: Supplementary file 1 — Additional file 1. [file 12885_2020_7459_MOESM1_ESM.docx]

| **Parameter** | **Classification** | **Distribution** | **Impact on CSS**  **Hazard ratio [95%-CI]** | **P-Value (logrank)** |
| --- | --- | --- | --- | --- |
| **Coronary artery disease**  NA = 4 | No  Yes | 85% (85/100)  11% (11/100) | Present: 1.68  95% CI [0.86-3.26] | 0.12 |
| **Chronic pulmonary disease**  NA = 4 | No  Yes | 73% (73/100)  23% (23/100) | Present: 1.31  95% CI [0.81-2.12] | 0.26 |
| **Diabetes**  NA = 5 | No  Yes | 81% (81/100)  14% (14/100) | Present: 1.35  95% CI [0.76-2.41] | 0.31 |
| **Arterial hypertension**  NA = 5 | No  Yes | 38% (38/100)  58% (58/100) | Present: 1.91  95% CI [1.22-2.98] | **0.0039** |
| **Atrial fibrillation**  NA = 5 | No  Yes | 85% (85/100)  10% (10/100) | Present: 1.00  95% CI [0.5-2.01] | 0.99 |

**Supplementary material**

Supplementary table 1: Univariate analysis of comorbidities affecting survival

Patient cohort was characterized according to listed parameters in the first column. Type of classification and distribution within the cohort as well as impact on survival including P-Value (logrank) is given for each parameter. Bold and underlined P-Values are meant to highlight those below 0.05. NA = cases where not for all patients baseline data was available.
